# Autogenous Trephine Core Sinus Lift and Simultaneous Implant Placement: A Case Report

**DOI:** 10.7759/cureus.87267

**Published:** 2025-07-04

**Authors:** G. Murali, Amit K Tamrakar, Mohammad Faisal, Saranjit S Bhasin, Rizwana Mallick

**Affiliations:** 1 Prosthodontics, Faculty of Dentistry, Jamia Millia Islamia, New Delhi, IND; 2 Oral and Maxillofacial Surgery, Faculty of Dentistry, Jamia Millia Islamia, New Delhi, IND

**Keywords:** autogenous bone trephine core, indirect sinus lift, osseointegration, osteotome, schneiderian membrane

## Abstract

This article describes a case of indirect sinus lift augmentation using autogenous trephine alveolar bone cores and simultaneous placement of dental implants in a highly resorbed posterior maxillary region. In comparison to the direct sinus lift approach, the procedure described in this case report is less invasive, results in reduced treatment time, and yields a more predictable outcome. This procedure involves a mucosal incision followed by surgical exposure of the alveolar bone, and then using a trephine drill to create a bone trephine core. The Schneiderian membrane is then gently pushed up along with the bone trephine core using an osteotome and mallet, followed by simultaneous dental implant placement. Although this technique is skill-sensitive, it results in successful osseointegration of the implant with fewer complications.

## Introduction

The common etiological factors for tooth loss in the posterior maxillary region are dental caries, periodontitis, trauma, or other diseases. When a natural tooth is lost in the posterior maxillary region, the residual alveolar bone generally resorbs rapidly. Due to this, the available bone height is limited for dental implant placement. In this case, implant placement and success are challenging due to several factors, such as maxillary sinus pneumatization, the presence of cancellous bone, limited surgical access, and poor visibility [1-4]. Therefore, when placing an implant in this site, a thorough understanding of these problems is required. This can help in formulating a treatment plan that will result in good initial stability of implants in this region, thereby facilitating early osseointegration of the implant with the bone. There are many methods to overcome these challenges, such as increasing bone height in this region through direct and indirect sinus lift procedures [3-7], using short and broad-diameter implants [8,9], placement of pterygoid implants [10], placement of zygomatic implants [11], and patient-specific machined or three-dimensional printed implants [12].

In direct sinus augmentation techniques, the sinus bone is exposed, drilled through, and the Schneiderian membrane is lifted, allowing for direct graft placement. This technique requires a longer period for prosthetic rehabilitation than the indirect sinus lift techniques [3-6]. Indirect sinus lift procedures can be performed with bone graft placement [3,6,7,13,14] or without bone graft placement [15-18].

In this case report, an indirect sinus lift technique utilizing an autogenous trephine alveolar bone core is presented, resulting in immediate implant placement and a significantly shorter prosthetic rehabilitation time.

## Case presentation

The present technique is a modification of Summer’s indirect sinus lift method, adapted to incorporate the use of a high-speed trephine drill and a sinus lift osteotome. This approach allows for a single-step implant osteotomy while using autogenous trephine alveolar bone core and osseodensification.

Surgical technique overview

The osteotomy is initiated using a trephine drill selected to be 1-1.5 mm smaller than the intended implant diameter. The trephine is advanced to a depth that is 1-2 mm short of the sinus floor, creating a cylindrical core of autogenous bone. This core remains attached apically and is not removed. A sinus lift osteotome, matched to the diameter of the planned implant, is then used to achieve two critical objectives, i.e., lateral bone compression (osseodensification) and sinus floor elevation. The osteotome compresses the lateral walls of the osteotomy, enhancing bone density around the implant site and increasing primary stability. Moreover, the gentle malleting of the osteotome drives the trephined bone core apically, elevating the Schneiderian membrane without perforation. The displaced bone core functions as an autogenous graft, supporting new bone formation within the sinus cavity. This combined technique promotes vertical bone gain and improved implant stability. The presence of the autogenous bone above the implant apex, along with lateral wall compression and the resultant blood clot, creates a biologically favorable environment for both osteoconduction and osteoinduction, facilitating accelerated bone regeneration compared to traditional sinus lift methods.

Diagnostic phase

**Figure 1:**
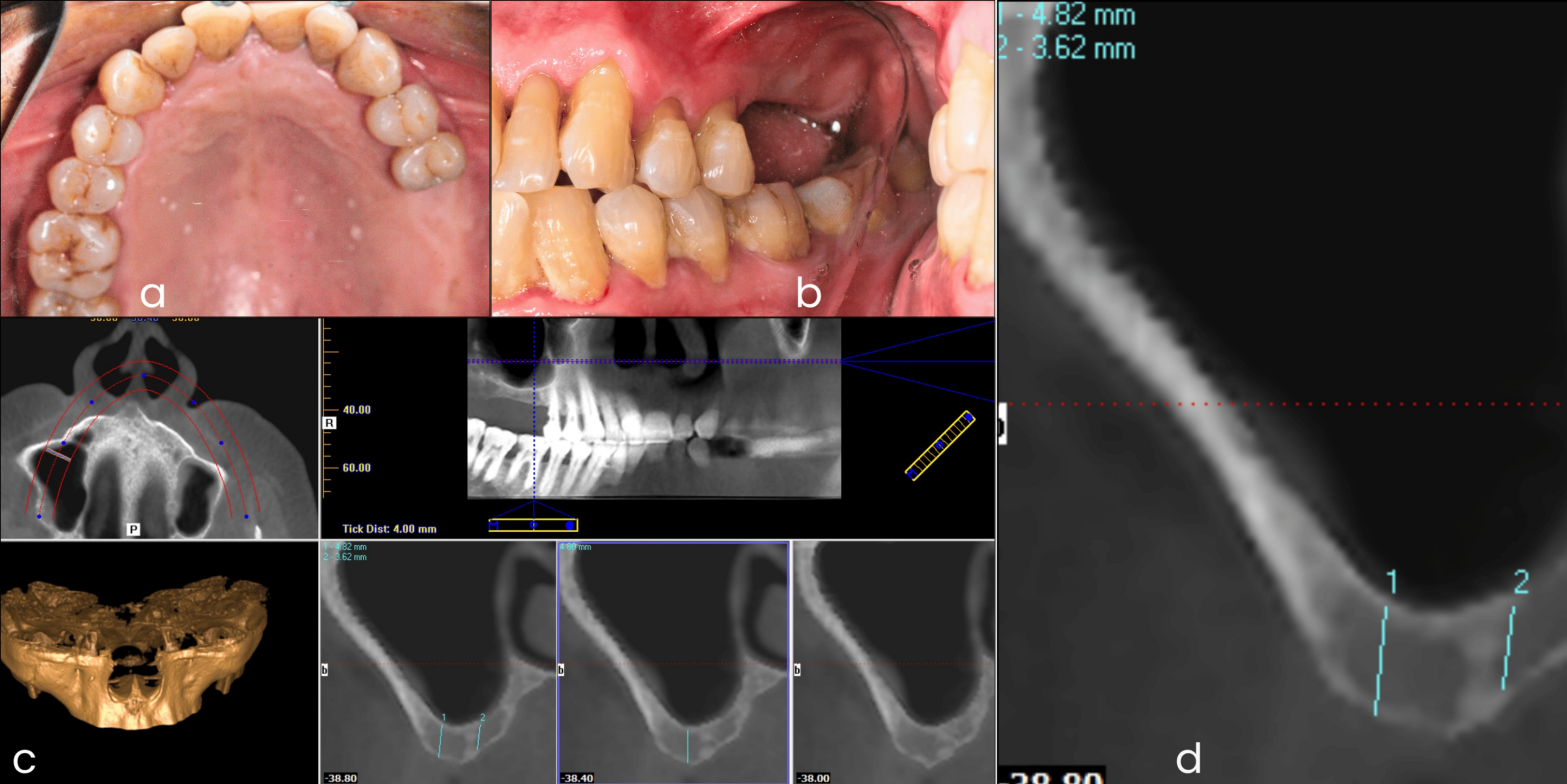
Photographs of the diagnostic phase including cone-beam computed tomography (CBCT) evaluation. (a) Intraoral maxillary arch occlusal mirror view. (b) Intraoral lateral mirror view. (c) Diagnostic CBCT image. (d) CBCT image assessment showing deficient bone height.

A 67-year-old male patient presented with a severely resorbed maxillary right posterior partially edentulous space and desired a fixed implant-supported prosthesis. A thorough clinical examination was conducted, and cone-beam computed tomography (CBCT) radiographic imaging was performed to assess bone quality and quantity. In the evaluation of CBCT images, the bone density was classified as D3 according to Misch’s classification [19]. The residual alveolar bone height was 4.82 mm, which was insufficient for conventional implant placement, necessitating sinus floor augmentation (Figure 1). Considering the patient’s request for a rehabilitation plan with a shorter duration, an autogenous trephine core sinus lift procedure and simultaneous implant placement were planned for the replacement of the missing maxillary right first molar and second molar. An oral prophylaxis procedure was planned before the surgical appointment.

Surgical phase

**Figure 2:**
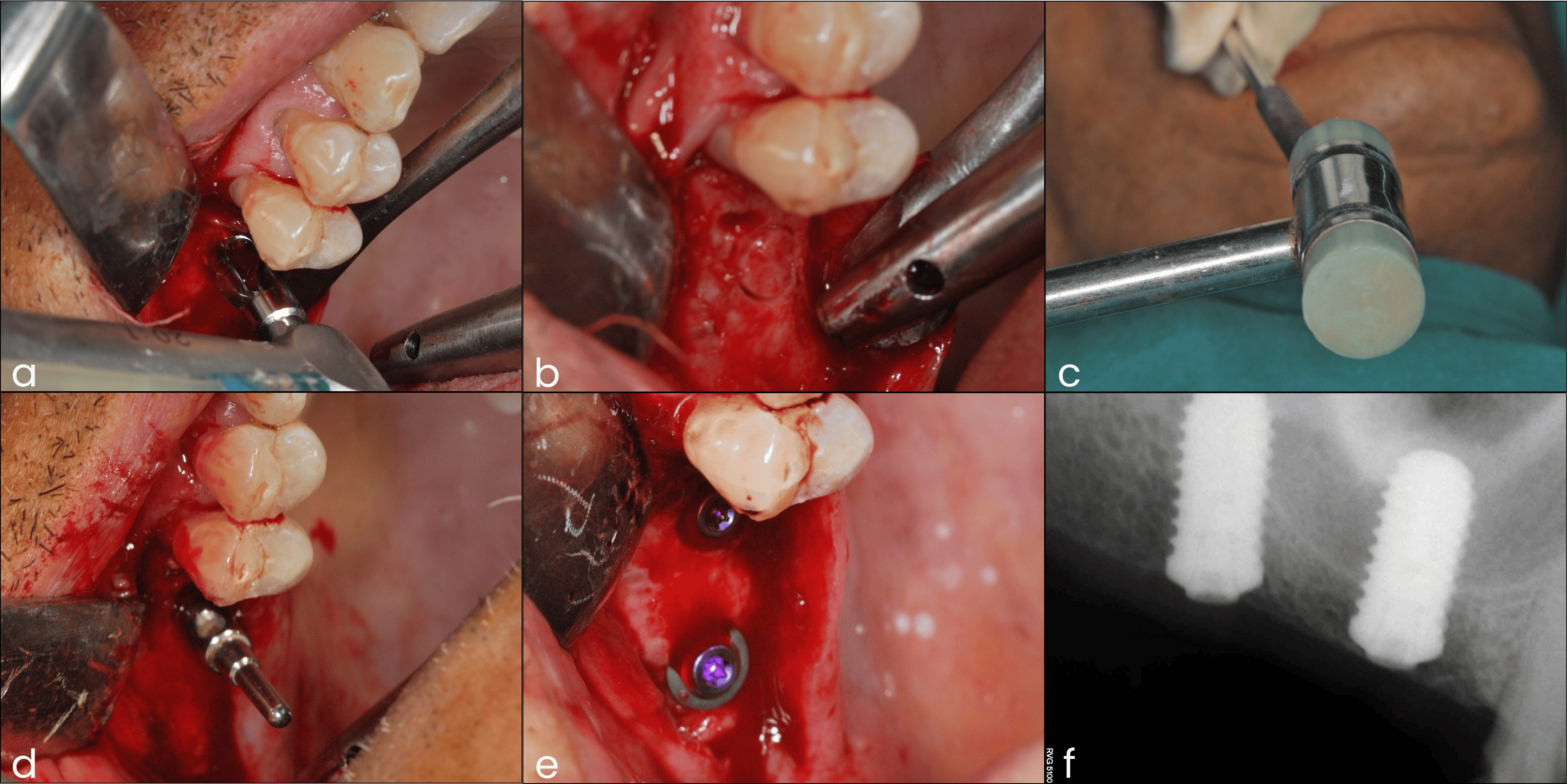
Photographs of the surgical phase and postoperative intraoral radiograph. (a) Trephine drill used to create an alveolar bone core. (b) Trephine bone core created. (c) Trephine bone core is apically pushed with the help of an osteotome and a mallet. (d) Implant positioning guide placed in the first implant osteotomy site. (e) Intraoral photograph after the placement of implants. (f) Postoperative intraoral radiograph.

Two days before the surgical appointment, the patient underwent oral prophylaxis. The modified trephine core sinus lift and simultaneous implant placement were performed under local anesthesia. A full-thickness mucoperiosteal flap was reflected to expose the maxillary edentulous ridge. At the site of the right maxillary first molar, a trephine drill with an outer diameter of 4.7 mm and an internal diameter of 3.9 mm was used to create a cylindrical bone core. Drilling was stopped 1-2 mm short of the sinus floor. Subsequently, a matched osteotome was gently tapped with a surgical mallet to drive the trephined bone core apically into the sinus cavity, effectively lifting the sinus membrane while ensuring the Schneiderian membrane remained intact. This process was repeated at the adjacent site of the missing maxillary right second molar region. An implant positioning guide was used to maintain near-parallel alignment of the implants during placement. Bredent Blue Sky self-tapping implants (5.5 mm diameter × 10 mm length) were then inserted into the osteotomy sites. Postoperative intraoral radiographs confirmed accurate implant placement and appropriate positioning of the autogenous trephine alveolar bone cores within the sinus cavity (Figure 2). Pain medications were prescribed, and the patient was advised to use a 0.2% chlorhexidine gluconate mouth rinse twice a day for two weeks.

Prosthetic phase

**Figure 3:**
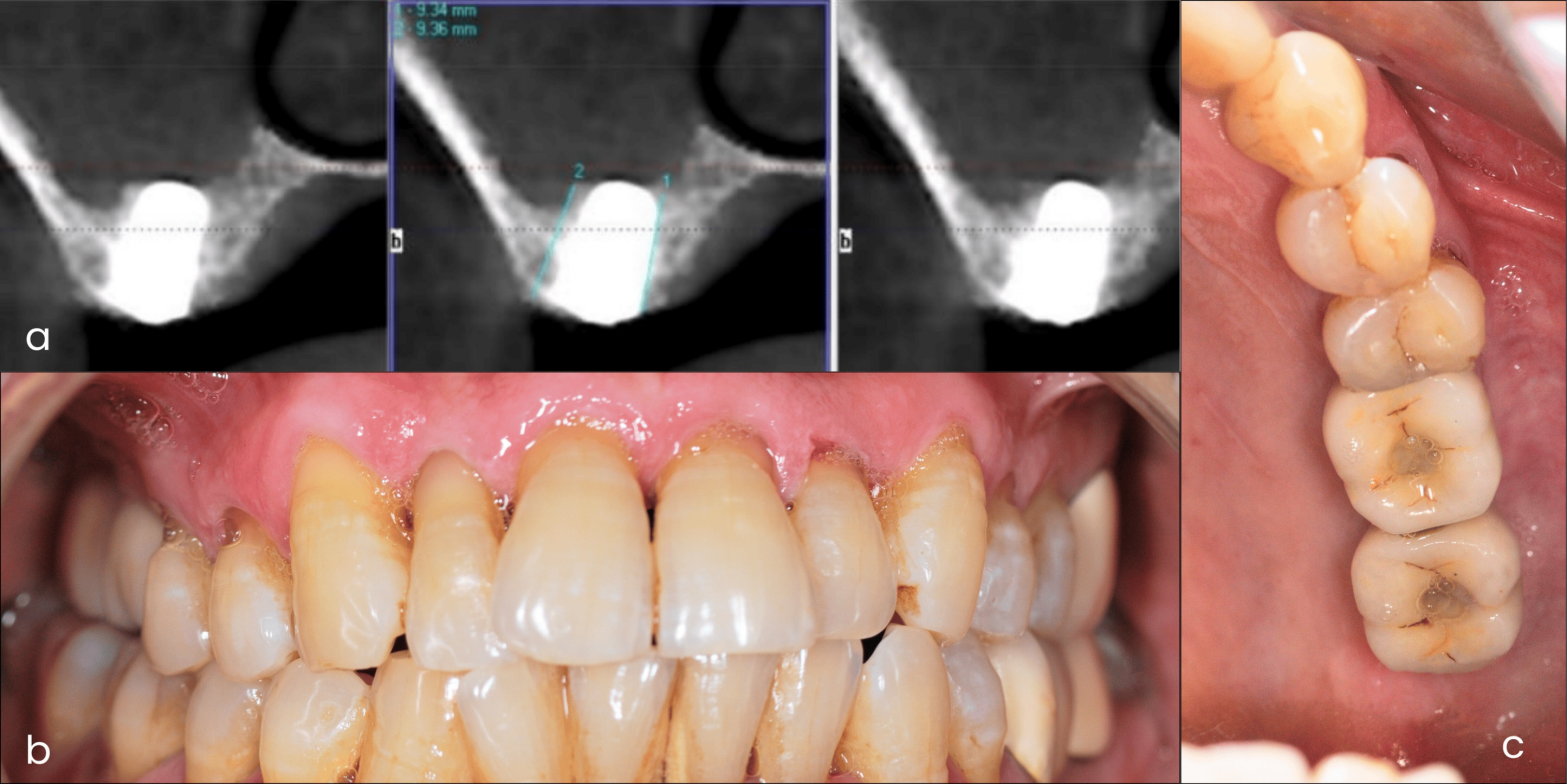
Five month postoperative cone-beam computed tomography (CBCT) image and postoperative intraoral photographs. (a) Five month postoperative CBCT image showing adequate bone formation. (b) Intraoral frontal view with the prosthesis in place. (c) Intraoral occlusal mirror view with the prosthesis in place.

The patient’s post-surgical recovery was uneventful. The implants were allowed to osseointegrate for five months. Good bone formation was achieved around dental implants, as confirmed by a five-month postoperative CBCT scan, which showed bone levels ranging from 9.34 mm to 9.36 mm. Subsequently, screw-retained, implant-supported crowns were placed, resulting in satisfactory esthetics and function (Figure 3). The patient also received oral hygiene instructions and was encouraged to maintain good oral hygiene practices.

Maintenance phase

**Figure 4:**
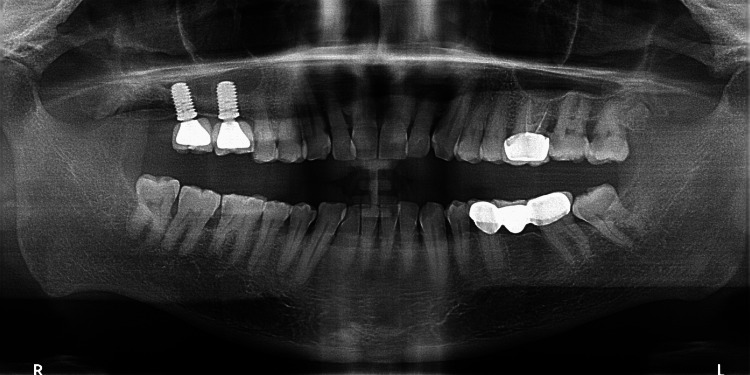
Two-year postoperative orthopantomogram.

The patient was recalled for follow-up examination after two years. The examination revealed healthy peri-implant soft tissues and stable implants with no mobility. The two-year follow-up orthopantomogram showed successful bone formation around the dental implants (Figure 4). Oral hygiene instructions were reinforced with special emphasis on the maintenance of peri-implant tissues.

## Discussion

The autogenous trephine core sinus lift technique involves raising a mucoperiosteal flap, creating a round cut in the residual alveolar bone with a trephine drill. Drilling below the sinus floor allows the trephine drill to create a cylindrical bone segment, referred to as a trephine core. Osteotomes are then used to elevate the membrane. For simultaneous implant placement, the trephine drill’s outer diameter should not exceed the implant’s core diameter to ensure proper implant thread engagement. A light pressure and an in-and-out drilling motion are recommended to prevent bone overheating [13,14,20].

This technique offers several advantages, including reduced treatment time, decreased morbidity, and improved patient satisfaction. Additional benefits include precise osteotomy, trephine size flexibility, and elimination of the need for a barrier membrane. It is particularly beneficial because it provides an autogenous graft from the exact osteotomy site, eliminating the need for separate harvesting. It combines bone preservation, precise membrane elevation, and simultaneous implant placement, thus reducing treatment stages and surgical trauma. The osteotomy is done in a single step, generating a trephine core bone block, leading to less heat generation in comparison to the use of multiple drilling procedures. The use of an osteotome also enables lateral compression and osseodensification without heat generation, which leads to enhanced primary stability and a lower likelihood of implant loss. Potential disadvantages include limitations due to trephine drill angulation and the need for careful execution of precise trephine osteotomy. However, careful patient selection, thorough preoperative planning, and precise surgical technique are essential for success [13-18,20].

During a sinus lift procedure, the potential complications include perforation of the Schneiderian membrane and loss of the dental implant into the maxillary sinus. Perforation can occur due to excessive force during sinus floor elevation [5,14,20]. Implant loss may result from significant sinus floor damage, inadequate implant stability, or severe membrane tearing [5,14].

The trephine core technique is most suitable in clinical conditions where there is a minimum residual bone height of 4 mm, with adequate crestal width. This bone height is sufficient to stabilize the implant and elevate the sinus floor with the trephine core. This may also be preferred when patients desire shorter treatment time with fewer surgical stages, and when autogenous bone is preferred and external harvesting is not feasible. It may be less ideal in cases where the residual bone height is less than 3 mm, in cases with highly pneumatized sinus cavities, or in cases with complex sinus anatomy where lateral access provides better visualization. In such cases, direct sinus lift techniques should be preferred [13-18,20].

## Conclusions

The autogenous trephine core sinus lift with simultaneous implant placement is a viable option for restoring severely resorbed posterior maxillary regions. It offers a biologically favorable, more predictable, and less invasive approach to sinus augmentation. This case report demonstrates the successful application of this minimally invasive procedure, resulting in satisfactory aesthetic and functional outcomes. Immediate implant insertion reduces treatment time and improves patient comfort. Careful patient selection, preoperative planning, and precise surgical technique are crucial to achieve the desired result. Long-term follow-up is necessary to assess peri-implant tissue stability and to ensure long-term implant success.
